# Methods, Applications, and Limitations of Somatic Maneuvers for the Modulation of Tinnitus

**DOI:** 10.3390/audiolres12060062

**Published:** 2022-11-18

**Authors:** Sumin Lee, Tae-Jun Jin, In-Ki Jin

**Affiliations:** 1 Department of Speech Pathology and Audiology, Graduate School, Hallym University, Chuncheon-si 24252, Republic of Korea; 2 Division of Speech Pathology and Audiology, Research Institute of Audiology and Speech Pathology, Hallym University, Chuncheon-si 24252, Republic of Korea

**Keywords:** somatic maneuvers, somatic tinnitus, somatic disorders

## Abstract

The modulation of tinnitus through somatic maneuvers is a well-documented phenomenon in tinnitus patients with somatic disorders. The purpose of this study was to review the methods, applications, and limitations of somatic maneuvers. First, 35 somatic maneuvers proposed by various research groups were summarized according to four body areas (jaw, head and neck, eye, and limbs), following which their applications and limitations were assessed. Although some studies have shown that somatic maneuvers can aid in screening for somatic tinnitus and may help alleviate symptoms with repeated practice, the limited number of studies and inconsistent results among studies make it difficult to draw definitive conclusions. Therefore, follow-up studies are required to overcome these limitations and determine whether the treatment of somatic disorders can also aid in alleviating somatic tinnitus.

## 1. Introduction

Tinnitus is characterized by the perception of sound in the ear or head regardless of the influence of external stimuli [1]. Generally, the reported prevalence of tinnitus in the adult population is approximately 10%, although this rate varies from 5.1% to 42.7% depending on the characteristics of the study group and region [2,3]. Some patients with tinnitus report severe pain, which may be accompanied by difficulty thinking and significant impairments in emotional regulation, sleep, hearing, concentration, and overall quality of life [4,5,6].

Tinnitus can be classified into various subtypes, and when the cause of tinnitus among the subtypes is related to the musculoskeletal system of the body rather than to the ear or hearing loss, it can be classified as “somatic tinnitus” [7,8,9]. Although not observed in all cases, one of the main characteristics of somatic tinnitus is that movement of or the application of pressure to the causative body part results in the modulation of tinnitus [8,10,11,12]. Such modulation has mainly been reported for the jaw, head and neck, eye, and limb [7,8,11,12].

The modulation of tinnitus due to somatic manipulation is a well-documented phenomenon in patients with tinnitus and somatic disorders [7]. Researchers have proposed various methods for identifying whether somatic manipulation can modulate tinnitus, which have been termed “somatic maneuvers” [7,11,12,13]. Several studies have suggested that these somatic maneuvers are useful when screening for somatic tinnitus and for identifying the true somatic disturbance leading to the condition [11,14,15], which increases the likelihood that the patient will experience the resolution of symptoms due to the prompt initiation of direct treatment [16,17,18]. Indeed, several groups have reported that alleviating somatic problems leads to tinnitus relief [16,17,18].

Although somatic maneuvers have been proven partially useful as screening tools for diagnosing somatic tinnitus, several limitations remain to be addressed. For example, the control of tinnitus in a specific body area does not always indicate somatic disorders in that area [11,14,19]. Thus, further research is required to develop methods for improving the reliability of somatic maneuvers. From a therapeutic perspective, some studies have suggested that repetitive somatic maneuvers can alleviate tinnitus via neuroplastic changes, although evidence that somatic maneuvers themselves lead to tinnitus relief is still lacking [12,16]. For somatic maneuvers to be useful in clinical practice, further studies are required to overcome the current limitations of diagnosis and treatment.

To promote a greater understanding of the value and limitations of conventional somatic maneuvers among researchers and aid in the development of novel methods, the present study aimed to review the methods, applications, and limitations of somatic maneuvers for various body parts (e.g., head, neck, arms, and legs).

## 2. Materials and Methods

**Figure 1:**
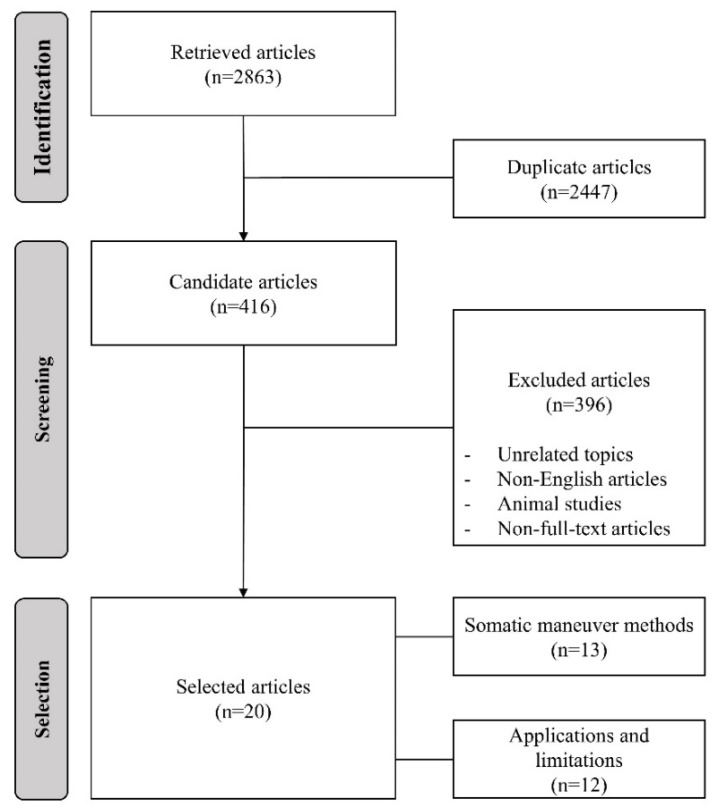
Flowchart of the article selection procedure.

A flowchart of the article selection procedure is shown in Figure 1. We searched Google Scholar for relevant articles using the following keywords: “somatic tinnitus”, “somatic tinnitus maneuver”, “somatic tinnitus treatment”, “somatic tinnitus disorder”, and “somatic modulation”. The search identified a total of 2863 articles, and 416 articles remained after excluding 2447 duplicates. Among the 416 articles, 396 that were not related to the topic of this review, non-English articles, animal studies, and non-full-text articles were excluded. We only reviewed the studies published in English, as this was the only non-native language common to all researchers, and we were concerned about misinterpreting papers published in other languages. Finally, 20 articles were selected for this review. Among them, 13 were reviewed for somatic maneuver methods, and 12 were reviewed for applications and limitations. Five articles were reviewed for both types of content.

## 3. Results and Discussion

In this study, the body parts related to somatic maneuvers were classified into four groups (jaw, head and neck, eye, and limb), and the somatic maneuver methods used to assess the modulation of tinnitus were described for each body part, as shown in Table 1.

### 3.1. Somatic Maneuver Methods

Generally, the modulation of tinnitus occurs with the movement of or application of pressure to body parts such as the jaw, head and neck, eye, and limbs (e.g., [7,14,21]). Somatic maneuvers require the patient to perform a specific motion under the guidance of the examiner, who then assesses whether modulation has occurred. Maneuvers may be performed with the patient or examiner applying pressure, and all somatic maneuvers are carried out for several seconds to ensure that the patient can recognize any change in tinnitus symptoms. When the modulation of tinnitus is observed after a specific maneuver, further testing is suspended until the tinnitus volume has returned to baseline [11].

Our review of somatic maneuvers related to jaw movement revealed that changes in tinnitus can be confirmed during the clenching of the teeth (maneuver 1) or when opening the mouth as wide as possible with or without the self-application of restorative pressure (maneuvers 2, 3). Similarly, the modulation of tinnitus can be assessed during the protrusion of the jaw with and without the self-application of restorative pressure (maneuvers 4, 5), following which additional assessments can be performed by having the patient slide their jaw to the left or right, with and without restorative pressure (maneuvers 6–9). Lastly, instructing the patient to retract their jaw (maneuver 10) can aid in identifying whether jaw movement can modulate tinnitus.

Several studies also described somatic maneuvers for the head and neck. First, with the patient’s head in a neutral position, the examiner can aid the patient in determining whether the modulation of tinnitus occurs during resistance to the self-application of force to the occiput, forehead, vertex, jaw, right temporal bone, and left temporal bone (maneuvers 11–16) in sequence. The patient then moves the head to position it as close to the shoulder as possible. If the movement is performed to the left, the left mastoid naturally contacts the left sternocleidomastoid muscle (maneuver 17). The patient then moves their head to the right, and the right mastoid naturally contacts the right sternocleidomastoid muscle (maneuver 18). Changes in tinnitus are then assessed by asking the patient to bend the neck forward, backward, left, and right (maneuvers 19–22) in sequence. Thereafter, modulation is assessed as the patient resists the maximal torsional force exerted by the examiner on the right zygoma, with the patient’s head turned to the right and left, respectively (maneuvers 23, 24). The final motion is performed with the patient’s head turned to the right and tilted to the left, allowing the examiner to resist the respective force applied to the left and right temples as much as possible (maneuvers 25, 26). Each maneuver is held for approximately 5 s to allow for the adequate assessment of tinnitus modulation.

Somatic maneuvers related to eye movements are also performed under the guidance of an examiner. The patient first moves their eyes horizontally (maneuver 27) and vertically (maneuver 28), followed by diagonal movement (maneuver 29). In each case, the maneuver is performed with a hold of at least 1 s in each direction.

Several studies also described somatic maneuvers related to the limb. The first movement involves locking the flexed fingers of the patient’s two hands together and pulling them apart as forcefully as possible (maneuver 30). Then, the patient spreads their right and left arms away from the center of their body in sequence (maneuvers 31, 32), followed by right and left shoulder abduction against resistance applied by the patient. The effects of hip movement on tinnitus are assessed with the patient in the supine position. First, each knee is bent to allow for the forward lifting of the leg (maneuvers 33, 34). Maneuvers 33 and 34 involve the flexion of the right and left hips against resistance applied by the patient, respectively. In this case, the examiner can assist the patient to ensure that body movements are performed correctly. Finally, the leg is lifted to the side to allow the patient to observe the modulation of tinnitus during the abduction of both hips against resistance (maneuver 35).

### 3.2. Applications and Limitations of Somatic Maneuvers

#### 3.2.1. Screening for Somatic Tinnitus

Several studies have suggested that the modulation of tinnitus can be observed during somatic maneuvers in the affected area in patients with somatic disorders accompanied by tinnitus [17,23,26]. Vielsmeier et al. (2011) compared the effects of somatic maneuvers on tinnitus in patients with and without temporomandibular disorders (TMDs) and the observed modulation of tinnitus in 50% and 21% of patients in these two groups, respectively, indicating a significant difference [17]. Ralli et al. (2016) reported that 79.67% of 310 patients with TMDs or neck dysfunction accompanied by tinnitus experienced the modulation of tinnitus when somatic maneuvers were performed in the same region [23]. In a study of 608 patients with tinnitus, Ward et al. (2015) also noted that patients with somatic tinnitus reported that the clear modulation of tinnitus had a higher rate of somatic disorders, such as TMDs, than those with non-somatic tinnitus [26].

However, other studies have reported that somatic maneuvers are not sensitive to somatic tinnitus. Sanchez et al. (2002) compared the tinnitus modulation rate in 68 patients with tinnitus who had craniomandibular disorders and 53 patients who had no symptoms of somatic disorders [24]. The modulation of tinnitus was observed in 67.6% of those with somatic symptoms and 62.3% of those without somatic symptoms, and there was no significant difference between the groups [24]. An et al. (2011) also reported no significant difference in the rate of somatic modulation between patients with somatic tinnitus (*n* = 24) and patients with sensorineural tinnitus (*n* = 21) [21]. Abel and Levine (2004) also noted that 60.5% of participants without tinnitus heard sounds similar to tinnitus during somatic maneuvers [20].

Although some studies have partially demonstrated that somatic maneuvers can be used to screen for somatic tinnitus, their application in this area may be limited due to inconsistencies in the results of previous studies (e.g., [17,21,23,24]). Therefore, additional research is required to identify the most reliable methods for somatic tinnitus screening using somatic maneuvers.

#### 3.2.2. Improvements in Tinnitus via Repetitive Somatic Maneuvers

Some researchers have focused on improving tinnitus using somatic maneuvers. Sanchez et al. (2007) proposed that training using repetitive somatic maneuvers can contribute to tinnitus relief based on the principle of neuroplasticity. In their study, 38 patients with tinnitus engaged in nine muscle contraction trainings twice a day for a total of 2 months. Although patients reported no changes in tinnitus during daily life, the loudness of tinnitus decreased during somatic maneuver training in 42.9% of participants [16]. In addition, Sanchez and Pio (2007) reported the resolution of symptoms in a patient with gaze-evoked tinnitus with a history of surgery for vestibular schwannosis who performed repeated eye movements for 14 weeks [27]. However, as only a limited number of studies have demonstrated improvements in tinnitus via training involving repeated somatic maneuvers, their value remains unclear at this time [28].

Several studies have suggested that the direct treatment of somatic disorders is effective in alleviating somatic tinnitus. Michiels et al. (2016) reported that 6 weeks of multimodal cervical therapy, including manual therapy, exercise therapy, and home exercise, resulted in significant improvements in 20 of 38 patients with cervical somatic tinnitus [29]. In addition, Wal et al. (2020) reported that 9 weeks of lifestyle guidance, facial treatment, and physical therapy significantly reduced tinnitus functional index scores in a cohort of 40 patients with temporomandibular somatosensory tinnitus [30,31]. Wright and Bifano (1997) described improvements in tinnitus in patients with TMDs who had been treated with a variety of approaches, including self-care guidelines to avoid muscle pain, splint and jaw stretching exercises, postural training and medication, and consultation with a psychologist. In their study, 52 of 93 patients reported tinnitus resolution, and 28 patients exhibited significant improvements after TMD treatment [32].

## 4. Conclusions

Currently, the etiology of somatic tinnitus seems unclear. Some studies have reported that diseases such as TMDs, cervical spine disorder, Paget’s disease of the bone, and restless legs syndrome are mainly accompanied by somatic tinnitus, but more studies are needed to confirm the relationship between these diseases and somatic tinnitus [17,33,34,35]. This study reviewed the methods, applications, and limitations of somatic maneuvers for assessing the modulation of tinnitus during movement or the application of pressure. Although some studies have shown that somatic maneuvers can aid in screening for somatic tinnitus and may help alleviate symptoms with repeated practice, the limited number of studies and inconsistent results among studies make it difficult to draw definitive conclusions. Therefore, follow-up studies are required to overcome these limitations and to determine whether the treatment of somatic disorders can also aid in alleviating somatic tinnitus.

## Figures and Tables

**Table 1:** Somatic maneuver methods for each body part.

Body Parts	No.	Method	References
Jaw	1	Clench teeth together	Levine et al. (2003, 2007) [13,19]; Abel and Levine (2004) [20]; An et al. (2011) [21]; Won et al. (2013) [14]; Kim et al. (2013) [22]; Ralli et al. (2016, 2018) [11,23]; Lee et al. (2020) [12]
	2	Open mouth with restorative pressure	Levine et al. (2003, 2007) [13,19]; Abel and Levine (2004) [20]; An et al. (2011) [21]; Won et al. (2013) [14]; Kim et al. (2013) [22]; Ralli et al. (2016, 2018) [11,23]; Lee et al. (2020) [12]
	3	Open mouth without restorative pressure	Levine et al. (2003, 2007) [13,19]; Abel and Levine (2004) [20]; An et al. (2011) [21]; Won et al. (2013) [14]; Kim et al. (2013) [22]; Lee et al. (2020) [12]
	4	Protrude jaw with restorative pressure	Levine et al. (2003, 2007) [13,19]; Abel and Levine (2004) [20]; An et al. (2011) [21]; Won et al. (2013) [14]; Kim et al. (2013) [22]; Ralli et al. (2016, 2018) [11,23]; Lee et al. (2020) [12]
	5	Protrude jaw without restorative pressure	Levine et al. (2003, 2007) [13,19]; Abel and Levine (2004) [20]; An et al. (2011) [21]; Won et al. (2013) [14]; Kim et al. (2013) [22]; Lee et al. (2020) [12]
	6	Slide jaw to left with restorative pressure	Levine et al. (2003, 2007) [13,19]; Abel and Levine (2004) [20]; An et al. (2011) [21]; Won et al. (2013) [14]; Kim et al. (2013) [22]; Ralli et al. (2016, 2018) [11,23]; Lee et al. (2020) [12]
	7	Slide jaw to left without restorative pressure	Levine et al. (2003, 2007) [13,19]; Abel and Levine (2004) [20]; An et al. (2011) [21]; Won et al. (2013) [14]; Kim et al. (2013) [22]; Lee et al. (2020) [12]
	8	Slide jaw to right with restorative pressure	Levine et al. (2003, 2007) [13,19]; Abel and Levine (2004) [20]; An et al. (2011) [21]; Won et al. (2013) [14]; Kim et al. (2013) [22]; Ralli et al. (2016, 2018) [11,23]; Lee et al. (2020) [12]
	9	Slide jaw to right without restorative pressure	Levine et al. (2003, 2007) [13,19]; Abel and Levine (2004) [20]; An et al. (2011) [21]; Won et al. (2013) [14]; Kim et al. (2013) [22]; Lee et al. (2020) [12]
	10	Retract jaw	Levine et al. (2003, 2007) [13,19]; Abel and Levine (2004) [20]; An et al. (2011) [21]
Head and Neck	11	Head in neutral position, with resistance to a force applied by the patient to the occiput	Levine (1999) [7]; Sanchez et al. (2002) [24]; Levine et al. (2003, 2007) [13,19]; Abel and Levine (2004) [20]; Won et al. (2013) [14]; Kim et al. (2014) [22]; Ralli et al. (2016) [21]; Lee et al. (2020) [12]
	12	Head in neutral position, with resistance to a force applied by the patient to the forehead	Levine (1999) [7]; Sanchez et al. (2002) [24]; Levine et al. (2003, 2007) [13,19]; Abel and Levine (2004) [20]; Won et al. (2013) [14]; Kim et al. (2014) [22]; Ralli et al. (2016) [21]; Lee et al. (2020) [12]
	13	Head in neutral position, with resistance to a force applied by the patient to the vertex	Levine (1999) [7]; Sanchez et al. (2002) [24]; Levine et al. (2003, 2007) [13,19]; Abel and Levine (2004) [20]; Kim et al. (2014) [22]; Ralli et al. (2016) [21]
	14	Head in neutral position, with resistance to an upward force applied by the patient to the mandible	Levine (1999) [7]; Sanchez et al. (2002) [24]; Levine et al. (2003) [13]; Abel and Levine (2004) [20]; Kim et al. (2014) [22]; Ralli et al. (2016) [21]
	15	Head in neutral position, with resistance to a force applied by the patient to the right temporal bone	Levine (1999) [7]; Sanchez et al. (2002) [24]; Levine et al. (2003, 2007) [13,19]; Abel and Levine (2004) [20]; Won et al. (2013) [14]; Kim et al. (2014) [22]; Ralli et al. (2016) [21]; Lee et al. (2020) [12]
	16	Head in neutral position, with resistance to a force applied to the left temporal bone	Levine (1999) [7]; Sanchez et al. (2002) [24]; Levine et al. (2003, 2007) [13,19]; Abel and Levine (2004) [20]; Won et al. (2013) [14]; Kim et al. (2014) [22]; Ralli et al. (2016) [21]; Lee et al. (2020) [12]
	17	Left mastoid attachment of the sternocleidomastoid	Won et al. (2013) [14]; Lee et al. (2020) [12]
	18	Right mastoid attachment of the sternocleidomastoid	Won et al. (2013) [14]; Lee et al. (2020) [12]
	19	Forward flexion of the neck	Ralli et al. (2016) [21]
	20	Backward flexion of the neck	Ralli et al. (2016) [21]
	21	Turn head to the right	Ralli et al. (2016) [21]
	22	Turn head to the left	Ralli et al. (2016) [21]
	23	With the head turned to the right, resist maximal torsional force applied by the examiner to the right zygoma	Levine (1999) [7]; Sanchez et al. (2002) [24]; Levine et al. (2003, 2007) [13,19]; Abel and Levine (2004) [20]; Won et al. (2013) [14]; Kim et al. (2014) [22]; Ralli et al. (2016) [21]; Lee et al. (2020) [12]
	24	With the head turned to the left, resist maximal torsional force applied by the examiner to the left zygoma	Levine (1999) [7]; Sanchez et al. (2002) [24]; Levine et al. (2003, 2007) [13,19]; Abel and Levine (2004) [20]; Won et al. (2013) [14]; Kim et al. (2014) [22]; Ralli et al. (2016) [21]; Lee et al. (2020) [12]
	25	With the head turned to the right and tilted to the left, maximally resist full force applied by the examiner to the left temple (left sternocleidomastoid)	Levine et al. (2003, 2007) [13,19]; Abel and Levine (2004) [20]; Won et al. (2013) [14]; Ralli et al. (2016) [21]; Lee et al. (2020) [12]
	26	With the head turned to the left and tilted to the right, maximally resist full force applied by the examiner to the right temple (right sternocleidomastoid)	Levine et al. (2003, 2007) [13,19]; Abel and Levine (2004) [20]; Won et al. (2013) [14]; Ralli et al. (2016) [21]; Lee et al. (2020) [12]
Eye	27	Movement of eye horizontally	Sanchez et al. (2007) [16]; Simmons et al. (2008) [25]
	28	Movement of eye vertically	Sanchez et al. (2007) [16]; Simmons et al. (2008) [25]
	29	Movement of eye diagonally to the upper and lower corners of the visual field	Simmons et al. (2008) [25]
Limb	30	Locking the patient’s flexed fingers of the two hands together and pulling them apart as forcefully as possible	Levine (1999) [7]; Sanchez et al. (2002) [24]; Levine et al. (2003) [13]; Abel and Levine (2004) [20]
	31	Right shoulder abduction against resistance applied by the patient	Levine (1999) [7]; Sanchez et al. (2002) [24]; Levine et al. (2003) [13]; Abel and Levine (2004) [20]
	32	Left shoulder abduction against resistance applied by the patient	Levine (1999) [7]; Sanchez et al. (2002) [24]; Levine et al. (2003) [13]; Abel and Levine (2004) [20]
	33	Flexion of the right hip against resistance applied by the patient	Levine (1999) [7]; Sanchez et al. (2002) [24]; Levine et al. (2003) [13]; Abel and Levine (2004) [20]
	34	Flexion of the left hip against resistance applied by the patient	Levine (1999) [7]; Sanchez et al. (2002) [24]; Levine et al. (2003) [13]; Abel and Levine (2004) [20]
	35	Abduction of both hips against resistance applied by the patient	Levine (1999) [7]; Sanchez et al. (2002) [24]

## Data Availability

Not applicable.
